# The influence of dog ownership on objective measures of free-living physical activity and sedentary behaviour in community-dwelling older adults: a longitudinal case-controlled study

**DOI:** 10.1186/s12889-017-4422-5

**Published:** 2017-06-09

**Authors:** Philippa Margaret Dall, Sarah Lesley Helen Ellis, Brian Martin Ellis, P Margaret Grant, Alison Colyer, Nancy Renee Gee, Malcolm Howard Granat, Daniel Simon Mills

**Affiliations:** 1 Institutite of Applied health research, Glasgow Caledonian University, Cowcaddens Road, Glasgow, G4 0BA UK; 2 School Of Life Sciences, University of Lincoln, Lincoln, LN6 7DL UK; 3 WALTHAM® Centre for Pet Nutrition, Waltham on the Wolds, Leicestershire, LE14 4RT UK; 4 Department of Psychology, State University of New York, Fredonia, NY 14063 USA; 5 School of Health Sciences, University of Salford, Salford, M6 6PU UK

**Keywords:** Elderly, Exercise, Sitting, Pet ownership, Physical behaviour, Dog walking, Human-animal interaction, ActivPAL

## Abstract

**Background:**

There is some evidence to suggest that dog ownership may improve physical activity (PA) among older adults, but to date, studies examining this, have either depended on self-report or incomplete datasets due to the type of activity monitor used to record physical activity. Additionally, the effect of dog ownership on sedentary behaviour (SB) has not been explored. The aim of the current study was to address these issues by using activPAL monitors to evaluate the influence of dog ownership on health enhancing PA and SB in a longitudinal study of independently-mobile, community-dwelling older adults.

**Methods:**

Study participants (43 pairs of dog owners and non-dog owners, matched on a range of demographic variables) wore an activPAL monitor continuously for three, one-week data collection periods over the course of a year. Participants also reported information about their own and their dog demographics, caring responsibilities, and completed a diary of wake times. Diary data was used to isolate waking times, and outcome measures of time spent walking, time spent walking at a moderate cadence (>100 steps/min), time spent standing, time spent sitting, number of sitting events (continuous periods of sitting), and the number of and of time spent sitting in prolonged events (>30 min). For each measure, a linear mixed effects model with dog ownership as a fixed effect, and a random effects structure of measurement point nested in participant nested in pair was used to assess the effect of dog ownership.

**Results:**

Owning a dog indicated a large, potentially health improving, average effect of 22 min additional time spent walking, 95%CI (12, 34), and 2760 additional steps per day, 95%CI (1667, 3991), with this additional walking undertaken at a moderate intensity cadence. Dog owners had significantly fewer sitting events. However, there were no significant differences between the groups for either the total time spent sitting, or the number or duration of prolonged sedentary events.

**Conclusions:**

The scale of the influence of dog ownership on PA found in this study, indicates that future research regarding PA in older adults should assess and report dog ownership and/or dog walking status.

**Electronic supplementary material:**

The online version of this article (doi:10.1186/s12889-017-4422-5) contains supplementary material, which is available to authorized users.

## Background

Physical activity (PA) is a well-recognised indicator and determinant of health [1] and more recently, sedentary behaviour (SB, sitting or lying with low energy expenditure whilst awake; [2]) has been identified as an independent risk factor for poor health [3]. Nonetheless, it is clear that whilst overall PA level decreases with age [4], older adults (>65 years) are also normally the most sedentary section of the population [5]. For adults, including older adults, national PA guidelines recommend 150 min per week of moderate to vigorous physical activity (MVPA; PA which raises the heart rate) [6]. Although a reduction in time spent in prolonged sitting is also recommended, there is no consensus about optimal sitting times. Maintaining appropriate levels of PA, or working towards this target at a later stage in life, has been shown to have significant health benefits for people with and without disease, and forms the focus for interventions targeting older adults. Higher levels of PA, including walking, are associated with improved health [7], reduced mortality, independent living, maintaining effective function and improved quality of life [8].

Dog ownership, and in particular dog walking as a feature of ownership, has been shown to be related to overall PA levels in a range of age groups. A meta-analysis of 29 studies conducted over 20 years examining the activities of Dog Owners (DO) and Non-Dog Owners (NDO) for a wide range of participants, including older adults, concluded that DOs walked more and were more physically active than NDOs [9], mostly from self-report PA measures. For example, post-menopausal female DOs were more likely to self-report 150 min per week exercise and less likely to be sedentary [10]. Older adult DOs (*n* = 330) engaged in more self-reported walking, not specifically intended as exercise (68 min/week), than non-pet owners (32 min/week) or non-dog pet owners (32 min/week) [11], but there was no significant difference between the groups in time spent walking for exercise (75, 62 and 52 min/week, respectively). The use of self-report means the robustness of these apparent effects in older adults can be questioned due to issues such as recall bias and social desirability bias*.* This can be a particular problem for studies investigating the effect of dog walking, as walking the dog may be a regular, planned activity that is easier to recall than other incidental PA, and also seen as something that owners should do, for example as a moral duty or to ensure the welfare of the animal. Objective measures of PA and SB provide opportunities to gain insight into both the intensity and pattern of PA and SB, allowing closer scrutiny of the potential relationship between dog ownership and health. Adult DOs who walked their dogs [12] had significantly longer time in MVPA (ActiGraph (ActiGraph Corp, Pensacola, FL, USA), 35 ± 24 min/day) and were more likely to meet PA recommendations (53%) than either NDOs (33 ± 24 min/day; 46%) or DOs who did not walk their dogs (27 ± 21 min/day; 33%). Older adults who walked their dog took approximately 1700 more steps (ActiGraph) than those who did not walk a dog [13].

A variety of monitors exist for objective measurement of physical activity and sedentary behaviour, with different strengths and weaknesses. The Actigraph monitor is worn at the hip and uses a threshold of low movement to identify SB, meaning that some activities undertaken while standing, such as washing the dishes, can be misclassified as sedentary tasks [14]. By contrast, the activPAL (PAL Technologies Ltd., Glasgow, UK) is worn on the front of the thigh and uses the static component of gravity to distinguish sitting and lying postures from standing, and is generally held as the gold standard for measuring SB [15]. The step count function of the Actigraph can underestimate total steps by as much as 40% at normal walking speeds (0.89 m/s [16]), whereas step count measured by the activPAL in older adults has been reported to be >99% accurate at similar speeds (≥0.67 m/s [17]). The activPAL is worn continuously, including overnight and during bathing (since it can be waterproofed), so that all PA and SB is measured, unlike the Actigraph, which is typically removed overnight and possibly at other times, increasing the likelihood that activity is missed.

Therefore, the aim of the current study was to use the activPAL monitor within a longitudinal design, in order to evaluate the association of dog ownership with both PA and SB in independently-mobile, community-dwelling older adults. Due to the potential for complex relationships between physical behaviour, dog ownership status and health, we chose to explore a range of health related physical activity and sedentary behaviour outcome measures. We hypothesised that owning a dog would be associated with increased physical activity (longer time spent walking, more steps taken) and reduced sedentary behaviour (less time spent sitting, less prolonged sitting, more sit-to-stand transitions).

## Methods

### Design

To assess the association of dog ownership on PA and SB of older adults, a case-controlled design was used where study participants (DOs and NDOs) were matched on a range of demographic variables. Using activPAL monitors, data was continuously sampled for three one-week data collection periods over the course of a year. This design was employed to reduce the risk of bias from drop-outs (e.g. if the third data collection period was always winter) and thus aimed to create a data set that was representative of a broad range of weather conditions.

### Ethical Approval

Full ethical approval was granted from the School of Life Sciences delegated authority of the University of Lincoln ethical approval committee, with further review and approval given by the WALTHAM animal welfare and ethical review board. All participants provided written, informed consent, and could withdraw from the study at any time without providing a reason.

### Sample size calculation

A sample size analysis indicated that 27 older adults per group would be sufficient to have 80% power to detect a difference in time spent walking of 30 min per day (as measured by activPAL), at a 5% significance level (Dall, utilising unpublished data from [18]). Allowing for a drop-out rate of 25%, the final target sample size was 40 per group.

### Study participants

Recruitment of participants took place between April 2013 and November 2014 until the target sample size was reached. A multi-point recruitment strategy was implemented using advertisement of the study on local radio and press, veterinary surgeries and other locations such as day centres, community groups, and local libraries. Participants were also given the opportunity to recommend others for the study although these participants were excluded from becoming the recommender’s matched pair to prevent social influences on outcome measures. Three distinct geographical regions in the U.K. (Lincolnshire, Derbyshire and Cambridgeshire, selected for convenience) were targeted concurrently.

Participants (both DO and NDO) needed to be aged 65 years or over, reside in a private residence in one of the three chosen geographical areas, have no scheduled health intervention(s) that could alter their PA during the time of data collection (e.g. scheduled surgery) and be able to walk unaided for a minimum of 10 min continuously. For DO, the latter criterion also applied to their dog(s). Participants were not excluded based on the presence or absence of specific mental or physical health conditions. Participants were assigned into matched pairs of DO and NDO based on age [+/− 5 years], gender, ethnicity, and socio-economic status [matching quintile of Townsend index [19] , derived from home postcode]. An additional matching factor of cat ownership was included since previous research provides conflicting evidence on the influence of this on physical activity [11, 20, 21]. At no stage during the study were participants made aware of any details of their matched pair.

### Data collection

PA and SB were objectively measured using a waterproofed activity monitor (activPAL™). The activPAL monitor has been validated for both postural classification and additional outcome measures in adults and older adults [15, 17].

Data collection took place between April 2013 and November 2014. For each participant, data were gathered during three data collection periods across a period of a year. Each data collection period lasted one week which occurred within one of three designated sampling intervals (March–June, July–October, November–February) to ensure data was collected across a range of seasons for each participant. Within a matched pair, data collection periods for the participants occurred within a four-week period. Initial data collection occurred throughout the year ensuring the first data collection period was not always in the same sampling interval.

Information used for matching was gathered at recruitment. At the first data collection period, participants provided further self-report information about themselves (see Additional file 1), including, height and weight (used to calculate BMI), chronic health conditions (self-reported presence/absence of at least one health condition), and distance that could be walked continuously (0.8, 1.6, 3.2, 4.8, 6.4, 8.0+ km; question asked in units of miles). At each data collection period, participants in the dog-owning group also provided demographic information about their dog(s), including age, type (pedigree, mixed breed, crossbreed), size (giant, large, medium, small, toy; examples were provided in the questionnaire), gender, and length of ownership. They also provided details of their role in caring for the dog(s), for example, what percentage of total responsibility they had for the dog, and whether the dog was usually walked on or off lead (see Additional file 1). In addition, when wearing the activity monitor, all participants completed a diary reporting the times they went to bed/got up, and the estimated times they fell asleep/woke up. This information allowed activity and SB related to waking times to be extracted from the activPALs.

### Study outcomes

Outputs from the activPAL monitors and information from the walking diaries were processed by a researcher (PD) blind to the groups. Data were downloaded and categorised using proprietary software (PALtechnologies version 7.1.18). Self-reported waking times from the diary were used in a hierarchical manner [(a) estimated sleep/wake times; (b) reported bed/get up times; (c) visual inspection] to isolate waking activity data. When diary data was not available, a second independent (blinded) researcher (BS) visually reviewed the activPAL output and estimated the waking period each day from the first and last activity in the day.

Outcome measures were calculated for the waking day via the event output from the activPAL monitor using a custom Excel macro. An event is defined as a continuous period of a single posture or activity [22]. Waking and sleep times were used exactly as recorded. Any event crossing the wake/sleep time was cut at that point, and only the part within the waking day was included in analysis. PA outcomes were the time spent walking, the time spent walking with a cadence of over 100 steps/min (equivalent to MVPA [23]), the number of steps taken and the time spent standing. SB measures (see [24]) were time spent sedentary, number of sedentary events, and the number and time spent sitting in prolonged sedentary events (> 30 mins). Although the duration of the waking day may have varied within and between participants, the proportion of the waking day engaged in an activity were not used for analysis, because choices, such as time of getting out of bed, may have formed an integral part of the lifestyle of participants. Finally, a binary outcome measure based on adherence to current PA guidelines for older adults (150 min per week of moderate PA [6]) was calculated using the total time spent walking at a moderate cadence across the data collection period. A pro rata threshold for duration of moderate activity was created based on the number of days of data assessed [i.e. 150 min per week* (number of days of assessment/7)], and participants were judged to have met the guidelines if they exceeded this threshold. This outcome measure was calculated separately for each data collection period for each participant.

### Statistical analysis

The same blinded researcher who undertook the data processing performed the statistical analyses. Data from a data collection period for a given participant was included in the analysis if there were at least three waking days at that data collection period. Pairs of participants were included in analysis if there were data from at least one data collection period for each participant in the pair. Baseline demographic variables and matching characteristics and hours awake during the day were compared between groups using paired *t*-tests or related samples Wilcoxon signed ranks tests as appropriate. Linear mixed effects models, with dog ownership as a fixed effect and a random effects structure of data collection period nested in participant nested in pair, were conducted to assess the effect of dog ownership on all physical activity and sedentary behaviour outcomes. For the proportion of individuals meeting pro-rata PA guidelines, a generalised linear mixed effect model was performed, with binomial distribution and logit link function, using the same random and fixed effects structure. Continuous data were log_10_ transformed as required based on inspection of the residuals, checking for assumptions of normality and constant variance. Where significant by a likelihood ratio test, the residual variance was weighted by dog ownership group. Mixed effects models using restricted maximum likelihood allow for estimation in the presence of missing data, therefore no imputation of data was deemed necessary. The effect of data collection period (time through study) on the measures was explored as both additive and multiplicative interactions with group. The model was not significantly improved by their inclusion (as tested by likelihood ratio tests) for any outcome, and so data collection period was removed from subsequent analysis. The aim of this study was to assess differences between groups (dog owners and non-dog owners) using matched pairs of participant to account for variability between groups. Matching was extremely successful, and additional testing for confounders was not performed in the models. Data were analysed using R (version 3.3.1) with libraries *lme4*, *nlme* and *mutcomp*, and a *p*-value of 0.05 was used to indicate significance.

## Results

**Fig. 1:**
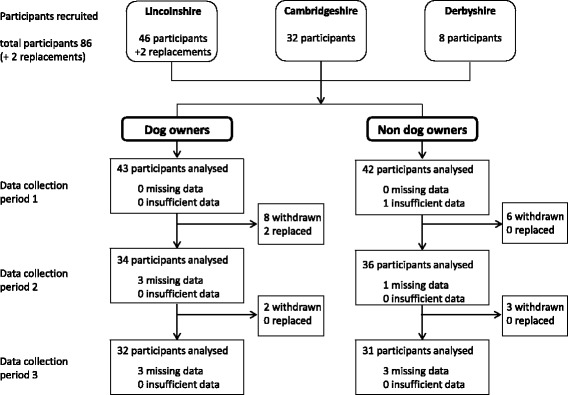
Flow diagram of participants progress through the study

Progress of the participants through the study is reported in Fig. 1. In total, 19 participants withdrew from the study, but in two of the pairs it was possible to replace the withdrawn participant with a different participant who was recruited and awaiting a match and who also met the matching criteria. The number of participants with data included in analysis was relatively similar across all sampling intervals (March–June, *n* = 77; July–October, *n* = 59; November–February, *n* = 82), with slightly lower levels of data collection over the summer.

Among those included in data analysis, compliance with wearing the monitor was excellent, with 92% (201/218 included datasets) having the full seven days of data. In total just 2% of potential days of data were not available for analysis. Most of the wake times used in the analysis (1310/1499 days, 87%) were derived from estimated wake and sleep times reported in the diary, with a further 11% (166 days) derived from diary reported bed times. Just 2% (34 days) of wake times were derived from the activPAL record.

Demographic data on participants are summarized in Table 1. Participants were white British (100%), mostly women (*n* = 54, 66%), aged (mean ± standard deviation (range)) 70 ± 4 (65–81) years, and most (*n* = 78, 91%) did not own a cat. Participants tended to live in less deprived areas, only 10 participants (12%) lived in the two most deprived quintiles using the Townsend Index. Although matched by Townsend Index quintile, pairs of participants lived (median (interquartile range) [range]) 13 (23) [0–60] km apart. The mean BMI of participants indicated they were overweight (25.6 ± 4.0 kg.m^−2^) ranging between underweight (18.9 kg.m^−2^) and obese (35.8 kg.m^−2^). Just over a third of participants reported having one or more chronic health condition at the start of the study, these included cardio-vascular and arthritic conditions, osteoporosis and visual impairment. The mean self-reported distance that participants could walk without stopping was 6.3 ± 2.4 km. The matching of pairs used in this study was extremely successful, the two groups were identical for all categorical outcomes (gender, ethnicity, socio-economic status, and cat ownership), and were not significantly different for the age at start of study, *p* = 0.560. Additionally, there were no statistical differences between groups for other demographic characteristics, BMI (*p* = 0.612), number of participants with a chronic health conditions (*p* = 0.821), or self-reported walking distance (*p* = 0.564).

**Table 1:** Participant characteristics by dog ownership status

	*N*	Dog Owners (*n* = 43)	Non dog owners (*n* = 43)
Match Characteristics			
Gender (*N* female, %)	43	27 (63%)	27 (63%)
Age at start of study (years)	43	69.9 (± 4.1)	70.2 (± 4.1)
Ethnicity (*N* White British)	43	43 (100%)	43 (100%)
Cat ownership (*N* yes)	43	4 (9%)	4 (9%)
Townsend Index	43		
1 (least deprived)		11 (26%)	11 (26%)
2		15 (35%)	15 (35%)
3		12 (28%)	12 (28%)
4		4 (9%)	4 (9%)
5 (most deprived)		1 (2%)	1 (2%)
Other Characteristics			
Distance between homes (km)	42	12.8 [22.8]	
BMI (kg.m^−2^)	43	25.4 (± 4.3)	25.8 (± 3.7)
Participants reporting a chronic health condition at start of study	38/41	14 (37%)	15 (37%)
Self-reported continuous walking distance (km)	38/37	6.1 ± (2.2)	6.5 ± (2.6)

Data are reported as number (%), mean (± standard deviation) or median [interquartile range].

Information on dogs owned was missing for two DOs. The remaining 43 DOs in this study owned 61 dogs, in total. Most DOs (*n* = 41, 95%) owned either one (*n* = 31, 72%) or two (*n* = 10, 23%) dogs, while the other two DOs owned four and six dogs. About half of the DOs reported that they were solely responsible for care (*n* = 17, 40%) and exercise (*n* = 23, 53%) of their dog(s). Of those DOs who shared responsibility for their dog(s), only a small number reported providing less than half of that care (*n* = 2) and exercise (*n* = 1). About half the dogs were female (*n* = 31, 49%), and most (*n* = 46, 75%) were neutered. Dogs were aged 7.7 ± 3.7 (0.3–15.0) years, and had been owned for most of their life 6.0 ± 3.7 (0.2–15.0) years. Most of the dogs owned were pedigree (*n* = 42, 69%), and the dogs were spread across a range of sizes (*n* = 20, 33% toy and small; *n* = 23, 38% medium; *n* = 18, 30% large and giant). Just under half of the dogs (*n* = 29, 46%) were usually walked on the lead.

Four outcome measures were successfully log_10_ transformed prior to analysis, and six benefitted from a model allowing heterogeneity of dog ownership group variances (Table 2). Participants were awake for 16.3 ± 1.0 (12.8–18.8) hours per day, with no significant difference between groups (*p* = 0.797).

**Table 2:** Physical activity and sedentary behaviour of dog owners and non-dog owners

	Dog owners	Non dog owners	Difference (DO - NDO)	*p*-value
Number of steps [/day] ^a,b^	10,030 (9063, 11,101)	7269 (6548, 8069)	2762 (1667, 3991)	<0.001
Time walking [min/day] ^a,b^	119 (109, 131)	96 (88, 106)	23 (12, 36)	<0.001
Time walking at a moderate cadence [min/day] ^a,b^	32 (23, 43)	11 (8, 15)	21 (12, 34)	<0.001
Time standing [hours/day] ^b^	4.44 (4.13, 4.75)	4.35 (4.04, 4.66)	0.09 (−0.22, 0.40)	0.566
Time sedentary [hours/day]	9.94 (9.54, 10.35)	10.25 (9.84, 10.66	−0.31 (−0.75, 0.13)	0.163
Number of sedentary events [/day] ^a,b^	44 (41, 47)	52 (48, 57)	−8 (−12, −5)	<0.001
Time in prolonged sedentary bouts [hours/day] ^b^	5.89 (5.39, 6.39)	5.45 (4.94, 5.96)	0.44 (−0.05, 0.93)	0.081
Number of prolonged sedentary events [/day]	5.82 (5.43, 6.22)	5.75 (5.35, 6.15)	0.07 (−0.38, 0.52)	0.756

Estimated group means. Data are presented as mean (95% Confidence interval), difference in means (95% Confidence Interval of difference) between groups (dog owners – non dog owners) and *p*-value of comparison. For outcome variables that were log_10_ transformed, differences in means have been calculate from fold changes.

*DO* dog owners, *NDO* non dog owners.

^a^data log^10^ transformed prior to analysis

^b^model allowed hetergeniety of do ownership group variances

DOs walked for significantly longer than NDOs overall and at a moderate cadence (Table 2). Consequently, DOs took significantly more steps than NDOs. The difference between the groups in time spent walking at least at a moderate cadence, of 21 min with 95%CI (12, 34 min), was similar to the difference in total time walking, 23 min with 95%CI (12,36 min), suggesting that the additional walking performed by the DOs was at a moderate cadence. Across all three data collection periods, significantly more DOs (87% (95%CI 61, 96)) than NDOs (47% (95%CI 19, 77)) met physical activity guidelines of 150 min of moderate activity per week (OR 75 (95%CI 3, 2167), *p* = 0.015). There was no significant difference between groups in time spent standing (Table 2). DOs and had fewer sedentary events, however there were no significant differences between the groups for time sedentary in total, the number or the duration of prolonged sedentary events (Table 2).

## Discussion

In this study owning a dog indicated a large, potentially health improving effect [25]: on average 20 min of additional time spent walking and 2700 additional steps per day with this additional walking undertaken at a moderate cadence (≥100 step/min). Indeed, the size of the difference may be sufficient to meet PA guidelines on its own (22 min of MVPA every day would achieve 150 min of MVPA per week). It is not surprising, therefore, that DOs were more likely to meet PA guidelines (87%, 95%CI 61, 96) than NDOs (47%, 95%CI 19, 77). Additionally, older adult DOs had 8 fewer sedentary events on average, but there was no difference in time spent sitting in total, in prolonged sedentary behaviour, or in time spent standing between the groups.

Previously reported group differences in MVPA in adults and adolescents, based on use of the ActiGraph were statistically significant, but often small (~2 min/day; [12, 26]). A small difference of 2 min/day is unlikely to have a large impact on health. The current study also found a larger difference in total step count (2700 steps) associated with dog ownership, than the difference found in the only other comparable study (1700 [13]). Although differences in total step count on their own do not provide information about the intensity at which they were taken, the PA guidelines could be achieved by taking 2200 steps per day (22 min per day, at a moderate cadence of 100 steps/min). Both studies, therefore, indicate a meaningful increase in walking due to dog ownership. In general, the NDOs in this group of older adults (7200 steps/day; 96 min/day walking) were within, but towards the top of, normal ranges, while the DOs (10,000 steps/day, 119 min/day walking) often exceeded usual ranges of PA compared to the general population of community dwelling older adults, (30–60% meeting guidelines, measured using self-report and ActiGraph [27, 28]).

Although not-significant, this study demonstrated a reduction in objectively measured SB of 19 min associated with dog ownership. Although there are no firm guidelines on SB and health, a dose relationship is apparent [3]. For example, a 1 h reduction in self-reported SB was equivalent to a 3% reduction in mortality in older women [29]. The scale of the difference associated with dog ownership in this study (19 min less time spent sedentary per day), is therefore likely to have a small positive influence on health. The only other study to assess objectively measured SB, used the ActiGraph and found a smaller and non-significant reduction in SB of 7 min/day in adolescents associated with household dog ownership [26]. Differences in measured sedentary behaviour may be due to different monitors used (assessing low movement versus posture), measurement protocol (removed overnight versus reported sleep), or differences in population behaviour (adolescents versus older adults). Of particular interest, is the composition of activity over a 24-h day. Given that the duration of a day is constant, a change in time spent in one type of activity, must result in a consequent and opposite change in time spent in other activities. In this study, there was little difference between the groups in time spent standing or time spent asleep, which implies that the reduction in time spent sedentary for the DOs (19 min reduction) was transferred into time spent in MVPA (21 min increase). Although the inter-relationship of sleep, SB and PA across the day is complex and under-researched [30], recent isotemporal time substitution analysis indicates that transfer of time spent sedentary to time spent in MVPA provides the maximum potential benefit to health [31].

Dog ownership represents a complex behavioural relationship between the DO, the dog(s), and other members of the household (including other pets). Owning a dog does not necessarily mean that an individual either cares for, or walks with, the dog. In some studies, dog walking, as opposed to dog ownership is assessed (e.g. [13]), and the general consensus in the literature is that it is dog walking, rather than dog ownership, which positively influences PA. In this study, the factor distinguishing groups was dog ownership, but we also assessed levels of self-reported caring for the dog. Most of the DOs in this study reported having sole responsibility for care of and walking the dog, with only one DO reporting less than 50% responsibility for walking the dog. A further factor that may influence the relationship of dog ownership and dog walking is the number and type of dog(s) owned. Factors which may influence this include gender, age, size [32, 33], breed, neuter status, temperament, energy [32] and behaviour of the dog [33, 34]. Theoretically, walking dogs on the lead may involve the DO walking slowly and stopping frequently, or dogs may get additional exercise when not on the lead. These factors should however be explored in more depth in future.

Although this study provides the best quality data to date on the effects of dog ownership on PA, there are several potential limitations to consider. Participants were volunteers and so may have been more physically active than the general population, so the results may not be completely generalisable. Any potential bias in the volunteers would apply to the NDOs as well as the DOs, and both groups had similar levels of health for the aspects we assessed, and so the results are valid for indicating the magnitude of effect. It should also be acknowledged that few volunteers were recruited from the lowest quintile of SES, and all volunteers had white British ethnicity, which may also limit the applicability of the findings to wider contexts. We did not assess the effect of confounders within our statistical models, however the matching between pairs of participants was excellent for common confounders, and therefore this is unlikely to have had a large influence on the difference between groups. The study was only powered to be able to detect changes in physical activity, and it is possible that a larger study would have detected a significant difference in sedentary behaviour outcomes. The design of this study does not allow any inference to be made about whether more active people are likely to own dogs, or whether DOs become more active through owning a dog.

A considerable strength of the study was use of an appropriate objective monitor to measure PA and SB. The activPAL monitor is considered a gold standard for measuring SB [15], and is accurate for measuring step count in older adults at normal walking speeds [17]. The monitor is also able to assess MVPA, using a threshold of cadence generally held to be at a moderate level. Because this is applied to the average cadence across an entire walking event, regardless of length, this assessment avoids issues involved with dividing PA data into arbitrarily defined units [35]. Although the ActiGraph monitor is generally held to be a good measure of MVPA, thresholds derived from laboratory based calibration studies are required to derive time spent in MVPA from hip acceleration [36]. A variety of different thresholds exist [30], differing between adults [e.g. 12] and children [e.g. 26], which can limit the ability for comparison between studies. Compliance with monitor wear and study protocols was high, leading to 92% of participants having a full 7 days data, this probably reflects our individualized recruitment strategy, and the ease of monitor wear, compared to larger generic studies. This compares favourably with other studies, for example, in the 2003–2004 National Health and Nutritional Examination Survey, only 67% of returned monitors had at least 4 days of data available for analysis [4]. As the monitor was worn continuously, including overnight and during water-based activities we were able to assess all PA and SB undertaken throughout the week. Measuring participants three times throughout a year allowed a robust assessment of whether a difference in PA is apparent throughout the year. Previous cross-sectional research into the influence of dog ownership on PA has generally not matched participants, instead breaking a group of recruited individuals into sub-groups based on their dog ownership status. In this study pairs of DOs and NDOs were matched on a range of basic characteristics (age, gender, ethnicity, SES, and cat ownership). Whilst there is a wide range of influences on both PA and SB behaviour, which may act on different levels (e.g. individual, environment, policy) [37], matching groups for some of the more basic characteristics has allowed the influence of dog ownership on PA and SB to be more effectively isolated.

## Conclusions

This study found that older adult DOs walked on average 20 min a day longer than NDOs. These results confirm previous studies where DOs reported more walking than NDOs, but also indicate that the additional walking of DOs was undertaken at a moderate cadence. On average, DOs met recommended public health guidelines (30 min/day of moderate PA), but NDOs did not. Owning a dog, may therefore motivate older adults to engage in appropriate levels of PA for health. Health promotion professionals could consider encouraging appropriate dog ownership, or shared care of a dog to promote PA in older adults. The scale of the influence of dog ownership on PA found in this study, indicates that future research regarding PA in older adults should assess and report dog ownership and/or dog walking status. It is important to note that even if dog ownership is not the focus of a piece of research examining PA in older adults it may represent an important explanatory factor which should not be ignored.

## Additional file

Additional file 1: Participant and Dog Information Questionnaire. The Participant and Dog information Questionnaire that was developed for the study. Participants in the dog ownership group completed a copy of the dog information section for each dog they owned. (PDF 166 kb)

## Abbreviations

BMI: Body mass index
DO: Dog owner
MVPA: Moderate to vigorous physical activity
NDO: Non dog owner
PA: Physical activity
SB: Sedentary behaviour
