# Supplementary material for: The influence of dog ownership on objective measures of free-living physical activity and sedentary behaviour in community-dwelling older adults: a longitudinal case-controlled study
Source: BMC Public Health. 2017 Jun 9;17:496. doi: 10.1186/s12889-017-4422-5 (PMC5465590; doi:10.1186/s12889-017-4422-5)
Supplement: Additional file 1: — Participant and Dog Information Questionnaire. The Participant and Dog information Questionnaire that was developed for the study. Participants in the dog ownership group completed a copy of the dog information section for each dog they owned. (PDF 166 kb) [file 12889_2017_4422_MOESM1_ESM.pdf]

We need to record a little bit of information about you and also about your dog(s) if you have any.

Section 1: About You

1. Participant ID:……………………………………………………………….

2. Gender: Male □ Female □

3. Age:………years……………..months…………………………………

4. Height:………..…………………………………………………………….

5. Weight:……………………………………………………………………

6. Are you a car owner? (please tick one)

Yes □ No □

7. Which of the following do you live in? (please tick one)
House □ Flat □ Bungalow □
Sheltered Housing □ Other□(please state)…………………

8. Do you regularly (i.e. weekly) undertake any leisure activities?
(e.g. swimming, bridge, bingo, rambling, yoga, meditation)
Yes No
□ □

If yes, please state what they are………………………………......

…………………………………………………………………………………

………………………………………………………………………………..

9. Please provide details of any current disorder(s) which impair your mobility

Participant and Dog Information questionnaire developed for: The influence of dog ownership on objective measures of free-living physical activity and sedentary behaviour in community-dwelling older adults. Mills D, Ellis S, Ellis B, Dall PM, Grant PM, Granat MH. Funded by an ISAZ/WALTHAM award 2012.

10. How many miles can you walk in one go? (please tick the highest of your capabilities)

0.5	1	2	3	4	5 or more
□	□	□	□	□	□

11. Do you have a long-standing physical or mental condition or disability (e.g. chronic heart disease, respiratory problems)?

Yes □ No □

If yes, please give details……………………………………………………

…………………………………………………………………………………………

…………………………………………………………………………………………

12. Have you ever had to give up a pet due to a health condition?

Yes □ No □

If yes, please give details (what and when)………………………

…………………………………………………………………………………………

…………………………………………………………………………………………

If you own a dog, please move on to Section 2. If you do not own a dog, please answer the following question.

13. (non-dog owners) Have you previously owned a dog?

Yes □ No □

If yes, please give details (what and when)………………………

…………………………………………………………………………………………

…………………………………………………………………………………………

Section 2: About Your dog(s)

Please complete a separate sheet for each dog you own.

Dog 1

1. Name………………………………………………………………………………

2. Gender (please tick one): Male ◻ Female ◻

3. Neutered (Please tick one) YES ◻ NO ◻

4. Age (to the nearest month)………………………………………..

5. What type of dog do you have?
Please tick one of the following.

◻ Pedigree (e.g. Labrador retriever)

◻ Mixed breed (e.g. Labradoodle, collie x Labrador)

◻ Crossbreed (i.e. parentage unknown or more than two breeds)

6. What size is your dog?
Please tick one of the following

◻ Giant (e.g. St Bernard)

◻ Large (e.g. German Shepherd)

◻ Medium (e.g. Springer Spaniel)

◻ Small (e.g. West Highland Terrier)

◻ Toy (e.g. Yorkshire Terrier)

7. Length of time you have owned him or her……………………

…………………………………………………………………………………..

8. Are you the sole carer for your dog? (please tick one)

YES ◻ NO ◻

If the answer is no, please give an approximate percentage of total responsibility for care (including exercising the dog) and a percentage of the time they are responsible for exercising the dog.

For example, if you are responsible for half of the dogs care but all of his exercise you would stipulate 50% for total care and 100% for exercise.

……..% total care

……..% exercise

9. Do you take part in any extra activities with your dog beyond walking outside of the home, for example agility, training classes, therapy work (please tick one)?

YES □ NO □

If Yes, please state what they are and how often you undertake them…………………..…………………………………………….

……………………………………………………………………………………………

……………………………………………………………………………………………

10. Do you take part in any dog related activities within the home environment, for example playing with the dog in the house or the garden (please tick one)?

YES □ NO □

If Yes, please state what they are and how often you undertake them…………………..…………………………………………….

……………………………………………………………………………………………

……………………………………………………………………………………………

11. Do you predominantly exercise your dog on or off lead for walks?

12. If there are times when you do not let your dog off lead when exercising, why is that? (Please tick as many as applicable)

□ Environment not suitable (e.g. too muddy, not secure, not allowed)

□ Not wanting my dog to interact freely with other dogs (e.g. for health reasons, behaviour reasons, for dog safety)

□ For reasons relating to my dog (e.g. poor recall, eating undesirable items, health reasons)

□ For reasons relating to myself (e.g. personal safety, health reasons)

13. Do you feel that this dog inhibits your lifestyle?
(For example your dog has dog become elderly and your exercise is restricted by your dog. Your dog may have separation anxiety and you can no longer go out without them or for short periods of time only.)

□ Matches my lifestyle

□ This dog restricts my lifestyle
